# Subsidy strategy of pharmaceutical e-commerce platform based on two-sided market theory

**DOI:** 10.1371/journal.pone.0224369

**Published:** 2019-10-31

**Authors:** Chenshuo Li, Zhe Huang

**Affiliations:** School of Business Administration, Shenyang Pharmaceutical University, Shenyang, Liaoning, China; Harbin Institute of Technology, CHINA

## Abstract

With the development of economic globalization and information technology, enterprises pay more attention to the sustainable development of their e-commerce. Under this trend, we study the subsidy strategy commonly used by pharmaceutical e-commerce platforms in two-sided market. Based on the two-sided market theory, we set up the two-sided user's utility function and formulate the subsidy strategy as the decision of platform profit optimization. We show that the platform chooses to subsidize consumers only if the net income from consumer is lower than the total revenue of drug retailers and platforms in each transaction; the maximum profit for platform increases with the intensity of the network externality. This study provides theoretical support and decision-making suggestions for the pharmaceutical e-commerce platforms to capture the market share, obtain higher profits and ultimately achieve the sustainable development goal.

## Introduction

With the development of network economy, two-sided market plays an increasingly significant role in the social economy, and the research on the two-sided market has been paid more and more attention by scholars. When two groups of participants in a market need to trade through an intermediate platform, and one party's profit depends on the number of participants on the other, the market becomes a two-sided market [1, 2]. With the advent of the era of network economy, the e-commerce platform which is different from the traditional market has become one of the important commercial activities of online transactions [3]. We conceptualize the e-commerce platform as a two-sided market with the main function of connecting consumers and retailers. E-commerce has greatly improved the benefits and efficiency of traditional commercial activities, attracting more other traditional retail industries (such as clothing, catering, medicine, etc.) to join the e-commerce platform, and the platform is highly competitive. The hallmark features of two-sided markets: the tendency for price movements across sides to be negatively correlated, and the number of users on one side increases, the other number of users increases [4, 5, 6]. In a two-sided market, the platform's profit depends on its ability to make a profit from users on two-side users of the transaction, mainly depending on the number of users on two-side of the platform, and the number of users on two-side of the platform is proportional to the number of transactions. Wei et al. [7] found subsidies for buyers as an effective way to keep retailers competitive. Therefore, the numerical estimation of the benefits associated with the cost of subsidies provides a basis for understanding the cost—benefit analysis of the subsidy strategy.

In this paper, the intermediate service organization—pharmaceutical e-commerce platform, which provides various services for consumers and pharmaceutical retail enterprises to provide transactions. The pharmaceutical e-commerce platform has the following characteristics: the transaction process is completed in whole or in part in the network environment, but the transaction subject must be certified by the authority real name; both sides users must have the qualifications to comply with the requirements of laws and regulations; the scope of the transaction, the conduct and manner of the transaction must be in full compliance with the requirements of laws and regulations. By cooperating with e-commerce platforms, drug retailers can obtain more consumer traffic than traditional business models, and to some extent translate into actual consumption and increase operating profit. Compared with other retail industries, China's pharmaceutical industry is developing rapidly, with the scale of pharmaceutical trade exceeding trillion yuan. However, the number and coverage of pharmaceutical e-commerce transactions are growing relatively narrowly, and the development of pharmaceutical e-commerce is slow.

**Fig 1:**
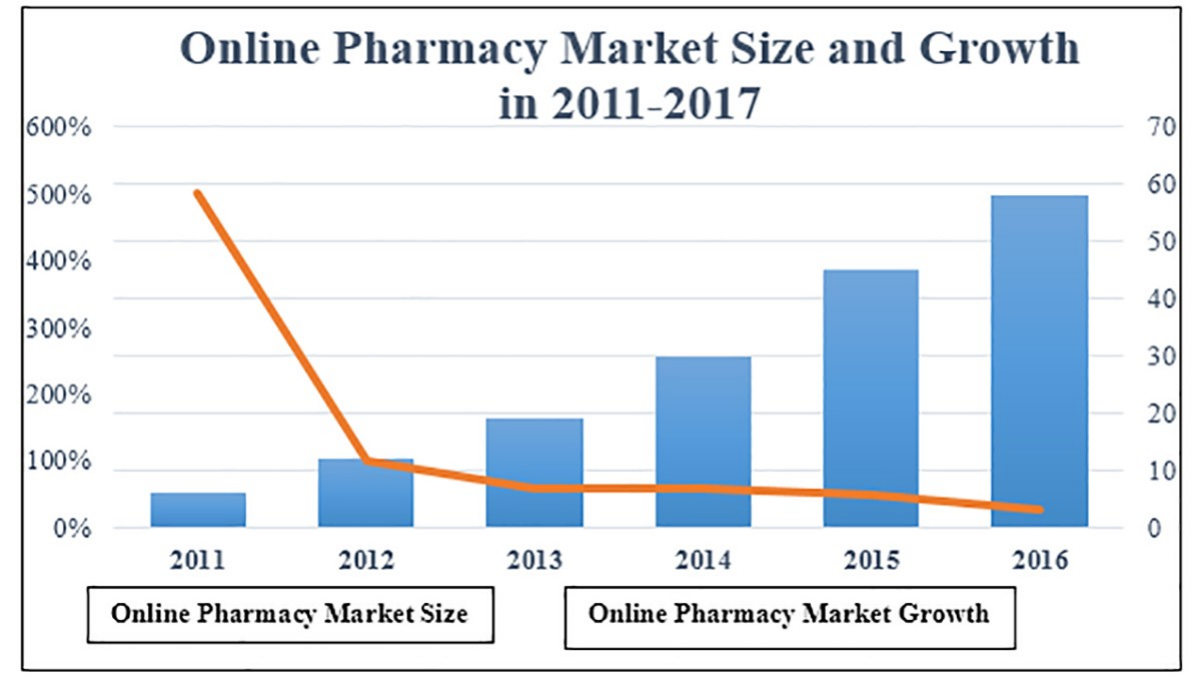
China online pharmacy market size and growth in 2011–2017.

In the new market environment, the online shopping model is gradually accepted and recognized by the public. The convenience and rapidity of the online shopping model make it the first choice for most consumers. Internet pharmaceutical e-commerce platform has a tremendous opportunity for development in the national policy support, pharmaceutical e-commerce booming and many other changes in consumer shopping habits, while also facing consumer acceptance, security of online transactions and a series of challenges. According to the monitoring data of China Pharmaceutical E-commerce Research Center [8], China's pharmaceutical e-commerce sales reached 47 billion yuan in, and large-scale pharmaceutical e-commerce platforms such as Ali Health, JD Pharmacy, Tmall Pharmacy came to the fore. The scale of online trading market has increased significantly, but the development of pharmaceutical e-commerce is relatively slow. Due to the particularity of drugs, the relevant legal system is not perfect, and drug insurance projects and other issues have not been resolved, so that the development of pharmaceutical e-commerce lags behind other e-commerce market, the market growth rate of online pharmacies slowed down year by year (Fig 1). In addition, netizens have low awareness of online pharmacies, and these factors have affected the sustainable development of pharmaceutical e-commerce.

With the rapid development of information technology, the Internet has been deeply integrated with economic and social fields, including pharmaceutical fields. In the fierce market competition, the pharmaceutical e-commerce platform seizes the market share monopolized by traditional pharmacies by means of ‘subsidy war’ and‘price war’, in order to obtain higher profits, adjust the platform supply and demand balance, and promote the sustainable development of the industry.

In this case, different subsidy strategies selected by the platform will produce different subsidy effects. Ali Health Pharmacy, for example, regularly issues full coupons or hosts a second half-price promotion to subsidize consumers, attract more and more consumers to Ali Health platform to buy drugs, and attracted a large number of drug retailers into the Ali Health platform. Because the sales cost of pharmaceutical e-commerce platform is lower than that of traditional retail pharmacy, a large number of circulation links are saved in the whole transaction process, which greatly reduces the operating cost of the pharmaceutical e-commerce platform. Therefore, in the stage of platform promotion, the platform can give consumers a certain subsidies without affecting profits, so as to seize the market and obtain higher profits. For the pharmaceutical e-commerce platform, in order to achieve the goal of sustainable development in the long-term market competition, a reasonable subsidy strategy is very important to it, the subsidy strategy can attract a large number of two-side users for the platform, and promote two-side users to trade on the platform. In view of this, it is worthwhile to study what kind of subsidy strategy should be adopted for the slow development of the pharmaceutical e-commerce platform at this stage, which has far-reaching practical significance for its sustainable development and profitability.

Based on the two-sided market theory, we build a game-theoretic model with network externality, and conduct an empirical study on the subsidy strategies of two-side users of pharmaceutical e-commerce platform.

There are three types of participants in the market, a pharmaceutical e-commerce platform, a group of potential consumers, and a group of potential drug retailers. The main purpose of our work is to study what kind of subsidy strategy is used by the pharmaceutical e-commerce platform to maximize the profit of the platform, and to explore the influence of the intensity of network externality on the platform subsidy strategy. On the one hand, we aim to theoretically explain the economic principle behind the subsidy strategy of the pharmaceutical e-commerce platform. On the other hand, we also hope to provide a good reference for the pharmaceutical e-commerce platform in the industry to design their subsidy strategies to efficiently incentivize users to join the platform, so that the platform can achieve sustainable development goals.

Our main findings are as follows. The platform chooses to subsidize consumers in order to maximize the profits of the platform, only if the net income of consumers in each transaction is lower than the total gains made by drug retailers and platforms in each transaction. When the platform aims at maximizing profits, the number of consumers and drug retailers participating in the transaction is proportional to the network externalities of two-side users. When the sales price of drugs rises, the platform tends to increase subsidies to consumers, reduce subsidies to drug retailers, and even charge drug retailers a certain transaction fees.

In the next section, we review some work related to subsidy strategies and two-sided market. In Section 3, we establish a game-theoretic model that describes the interaction among the pharmaceutical e-commerce platform, consumers, and drug retailers. The analytical results of the basic model are then presented in Section 4. We examine extensions about net income and network externality in Section 5 to draw more fruitful implications. Section 6 concludes the paper with discusses of results and future research.

## Literature review

In recent years, with the evolution of market structure, two-sided markets have become the frontier of scholars' research. Kim [9], Dou et al.[10], Shih et al. [11] and Zhang et al. [12], identified and analyzed platform management decisions based on two-sided market theory, and maximize platform profits by building a platform market model. Hagiu and Halaburda [13] studied the effect of different levels of information on the profits of two-sided platforms under monopoly and competition conditions. Lin et al. [14] examined the optimal two-sided pricing strategy of the platform while considering the seller's innovative decision-making and price competition. Howell [15], Emmanuel and Hagiu[16], Chao and Derdenger [17] shown that the strategic interactions between two-sided platforms depend not only on their price decisions, but also on whether the platforms are equally subsidizing one side of the market.

With the increasingly fierce competition in the market, it is particularly important for enterprises to take measures and have a long-term perspective in order to stand out in the fierce competition and achieve sustainable development. Subsidy strategy is the common price strategy of various industries at present, although the income of enterprises is not significant in a short period of time, but it has an important impact on the long-term sustainable development and profitability of enterprises [18]. By studying the impact of information at different levels on the profits of two-sided platforms, Hagiu and Halaburda [13] have shown that under monopoly and competition, platforms with more market forces benefit more because they can leverage and capture the responsiveness that leads to increased demand. Jin et al. [19] constructed two supply chain models, including manufacturers, consumers and governments, on the basis of which the optimal government subsidies and the optimal output and price of products were obtained through game theory and utility functions. Fan and Dong [20] explored and built an evolutionary game model including enterprises and consumers, discussed the choice of government subsidy strategies in low-carbon diffusion, and gave four kinds of government subsidy strategies.

In the era of network economy, the two-sided platform has greatly reduced the cost in the traditional trading process, in order to seize the market in the increasingly fierce competition, platforms frequent use of subsidy strategy to attract users from both sides to join the platform to trade. Under the influence of network externality, the more sellers on the platform, the platform will attract more buyers participate the platform to trade, the same, the more buyers on the platform, the platform will attract more sellers to the platform [21, 22]. The subsidy strategy of the platform to one side of the user can increase the number of users on this side, thus affecting the number of users on the other side [23, 24]. As an important supplement to the pricing strategy, subsidies directly affect the interests of both participants in the transaction process, aiming to create the correct incentives for each value chain participant. Caillaud and Jullien [25] believed that if the platform enterprises want to successfully gain market share and weaken competition, they must subsidize one side of the user. The subsidy between the two-sided platforms will inevitably reduce the profits of users on both sides, but only by losing the current small profits can expand the platform scale, bring more users and higher profits to the platform, and the subsidy strategy is the transition strategy of the early development stage of the two-sided platform.By assessing the effect of online travel agencies subsidizing consumer purchase guarantee policies, Bilotkach and Rupp [26] found that highly subsidized, low-priced customer guarantee policies are most popular with consumers and have a greater competitive advantage. Wei et al. [7] studied the effect of the buyer's subsidy strategy on the final utility of suppliers and buyers, and provided a basis for understanding the cost-benefit analysis of the buyer's subsidy strategy by numerically estimating the cost-related benefits.

Although the development of pharmaceutical e-commerce is fast, the research on pharmaceutical e-commerce platform is less in recent years. On the basis of in-depth study of the characteristics of China's pharmaceutical retailing, Zhang [27] established three kinds of pharmaceutical e-commerce platform model, which provides strategies for pharmaceutical enterprises to obtain more profits, and puts forward some suggestions for the development of pharmaceutical e-commerce platform. Based on the two-sided market theory, Wang et al. [28] studied and analyzed the pricing model of drug trading service platform and its influencing factors. The research shown that the main factors affecting the pricing of drug trading service platform include the level of market competition, network externality, marketing differentiation strategy of users on both sides of the platform and so on. In the previous research of less medical e-commerce platform, it is rare to find a research on the subsidy strategy of pharmaceutical e- commerce platform. Most scholars pay more attention to the development of other areas of the e-commerce platform, and lack attention to the early development of pharmaceutical e-commerce platform. Table 1 highlights the innovative breakthroughs of this paper in the field of two-sided platform by comparing the existing literatures on network externality, pharmaceutical e-commerce platform and subsidy strategy.

**Table 1:** A comparative overview of relevant literature research.

Research Orientation	Main research Contents
**Two-sided market theory**	Research on Platform Pricing Strategy by Constructing Model
**Subsidy strategy**	Subsidy strategy is an important marketing strategy for platform to occupy market share and attract consumers.
**Pharmaceutical e-commerce platform**	Influencing the pricing factors of platform and the development of platform
**This paper**	Research on subsidy strategy and influencing factors of pharmaceutical e-commerce platform based on two-sided market theory

Therefore, different from the previous research, considering the pharmaceutical e-commerce platform has a greater prospect for development, this paper studies the subsidy strategy of the pharmaceutical e-commerce platform in the context of the network economy era. On the basis of the existing research, based on two-sided market theory and using game theory method, this paper constructs a platform subsidy strategy model considering the maximization of platform profit and the utility function of both sides, and studies the subsidy strategy to be determined by the platform in order to obtain the maximum profit, under what circumstances the subsidy drug retailer or consumer and the subsidy level are affected by what factors, in order to provide theoretical support and decision suggestions for the pharmaceutical e-commerce platform in the development stage in grasping market share and obtaining higher profit.

## Model

Referring to the model of Ma et al.[29], Borcka and Wrede [30], we consider a market with two groups of participants, consumers and drug retailers, and a monopolistic pharmaceutical e-commerce platform which aims to maximize profits and provide platform services. Different from the traditional retail market, there is network externality in the two-sided market. To join the platform, consumers can obtain the positive network externalities brought by drug retailers, and the more drug retailers, the higher the utility of consumers. Similarly, drug retailers can gain positive network externalities from consumers by joining the platform, and the more consumers, the higher the profits of drug retailers. Let *M*_*C*_ and *M*_*D*_ be the potential maximum number of consumers and drug retailers in the market. When consumers enter the platform, they will get utility *v*(*v*>0) from the platform services. In each transaction, the consumer needs to pay the transaction cost *V*_*C*_ to obtain the revenue *R*_*C*_, and obtain the platform's fixed subsidy *S*_*C*_, which *R*_*C*_>*V*_*C*_. A consumer's utility for join the platform to participate in the transaction *U*_*C*_(*v*) is given by function (1):

$$U_{C}(v)=v+(R_{C}-P)N_{D}+\eta_{d}N_{D}-V_{C}+S_{C}$$ (1)

Among them, (*R*_*C*_−*P*)*N*_*D*_ indicates the net utility of the transaction to the consumer, and *η*_*d*_*N*_*D*_ indicates the network externality effect brought by the drug retailer to the consumer.

Drug retailers sell drugs priced at *P* to consumers on the platform through the pharmaceutical e-commerce platform. For every transaction completed, drug retailers can get *S*_*D*_ subsidy from the platform. The pharmaceutical e-commerce platform gains profits through revenue sharing with drug retailers, and drug retailers pay the revenue to platform in proportion to 1−*ω*(*ω*>0). The opportunity cost for drug retailers to participate in the transaction is *f*, and the drug retailers have to bear the operation cost *V*_*D*_ for each transaction. Same as above, the utility function of each drug retailer participating in the transaction is as follows:

$$U_{D}(f)=(\omega P-V_{D})N_{C}+\eta_{c}N_{C}-f+S_{D}$$ (2)

According to the principle of utility maximization, assuming that a consumer or drug retailer will join the platform if *U*_*C*_(*v*)≥0 or *U*_*D*_(*f*)≥0, respectively. Therefore, the undifferentiated utility function of consumers and the undifferentiated opportunity cost function of drug retailers are as follows:

$$\bar{v}=-(R_{C}+\eta_{d}-P)N_{D}+V_{C}-S_{C}$$

$$\bar{f}=(\omega P-V_{D}+\eta_{c})N_{C}+S_{D}$$

for simplicity of calculation, we assume that, *α*≡*R*_*C*_+*η*_*d*_−*P*, *b*≡*ωP*−*V*_*D*_+*η*_*c*_ that

$$\bar{v}=-\propto N_{D}+V_{C}{-S}_{C}$$ (3)

$$\bar{f}=bN_{C}+S_{D}$$ (4)

In order to maximize its own profits, the platform adopts the following three subsidy strategies for users on both sides: Strategy 1—the platform only selects subsidized consumers; Strategy 2—the platform only selects subsidized drug retailers; Strategy 3—the platform chooses to subsidize both users simultaneously. Equilibrium state, the users on both sides of the platform have a positive demand, participation in the transaction can obtain non-negative benefits.

A list of notations introduced so far is provided in Table 2.

**Table 2:** Variable parameter symbol description.

Hypothetical Variable and Meanings	
***D***	Drug retailers
***C***	Consumers
***N***_***D***_**, *N***_***C***_	Number of drug retailers and consumers involved in the transaction
***M***_***D***_**, *M***_***C***_	Number of potential largest drug retailers and consumers in the market
***η***_**d**_**(*η***_**d**_**>0)**	Intensity of network externalities brought by drug retailers to consumers
***η***_**c**_**(*η***_**c**_**>0)**	Intensity of network externalities brought by consumers to drug retailers
***v*(*v*>0)**	Usefulness of consumers entering the platform
***V***_***D***_**, *V***_***C***_	Transaction costs for each drug retailer and consumer
***R***_***C***_	Revenue earned by consumers on each transaction
***P***	Retail drug prices on the platform
***f***	Opportunity cost for drug retailers to participate in the transaction
***S***_***D***_**, *S***_***C***_	Subsidy level of platform to drug retailers and consumers
**1−*ω*(*ω***>**0)**	Proportion of revenue paid by drug retailers to platform

## Optimal solution analysis

In this section, we analyze the optimization problems of the platform. Through the above assumptions, we first derive the number of drug retailers and consumers involved in the transaction, and the subsidy level of platform to drug retailers and consumers. We then compare the maximum profits resulted from the three strategies and compare the most profitable strategies by Payoff Matrix. Finally, we find that the platform chooses the subsidy strategy 1 to maximize the platform profit without considering other factors.

We first analyze the drug retailers' and consumers' participation decisions. A customer with a utility of *v* will only participate in the transaction when the utility is not negative, the undifferentiated utility function of consumers is

$$\bar{v}=-\propto N_{D}+V_{C}{-S}_{C}$$

so the number of customers participating in the transaction is

$$N_{C}=M_{C}(1-F(\bar{v}))$$ (5)

The subsidy level for each customer is

$$S_{C}=F^{-1}\left(1-\frac{N_{C}}{M_{C}}\right)-\propto N_{D}+V_{C}$$ (6)

A drug retailer with an opportunity cost of *f* will only join the platform and participate in the transaction when the expected utility is non-negative, the undifferentiated utility function of drug retailers is

$$\bar{f}=bN_{C}+S_{D}$$

so the number of drug retailers participating in the transaction is

$$N_{D}=M_{D}H(\bar{f})$$ (7)

The subsidy level for each drug retailer is

$$S_{D}=H^{-1}\left(\frac{N_{D}}{M_{D}}\right)-bN_{C}$$ (8)

The platform shares transaction revenue with drug retailers, and adjusts platform demand through subsidy strategy to achieve the goal of maximizing its own profits. Assuming that the operating cost of the platform is zero, the maximum profit decision of platform choosing subsidy strategy can be described as:

$$Max\;\pi (S_{C},0)=(1-\omega )N_{C}N_{D}P-S_{C}N_{C}$$ (9)

$$Max\;\pi (0,S_{D})=(1-\omega )N_{C}N_{D}P-S_{D}N_{D}$$ (10)

$$Max\;\pi (S_{C},S_{D})=(1-\omega )N_{C}N_{D}P-S_{C}N_{C}-S_{D}N_{D}$$ (11)

For simplicity of calculation, assume *v*~*U* [0,*T*], *f*~*U* [0,*F*], then the number of consumers and drug retailers involved in the transaction can be simplified as follows:

$$N_{C}=\frac{M_{C}}{T}(T+\propto N_{D}-V_{C}+S_{C})$$ (12)

$$N_{D}=\frac{M_{D}}{T}(bN_{C}+S_{D})$$ (13)

Based on the above assumptions, it is possible to construct a Payoff Matrix that the platform selects to subsidize both sides of the user, as shown in Table 3.

**Table 3:** Payoff matrix of platform selection subsidies users on both sides.

Drug retailers	Consumers	
	Subsidy	No subsidy
**Subsidy**	*π*(*S*_*C*_,*S*_*D*_) = (1−*ω*)*N*_*C*_*N*_*D*_*P*−*S*_*C*_*N*_*C*_−*S*_*D*_*N*_*D*_	*π*(0,*S*_*D*_) = (1−*ω*)*N*_*C*_*N*_*D*_*P*−*S*_*D*_*N*_*D*_
**No subsidy**	*π*(*S*_*C*_,0) = (1−*ω*)*N*_*C*_*N*_*D*_*P*−*S*_*C*_*N*_*C*_	*π =* (1−*ω*)*N*_*C*_*N*_*D*_*P*

Through the payment matrix, we can clearly see that the platform has the greatest profit without subsidizing users on both sides, but the profit obtained in this case is only temporary. If the platform chooses not to subsidize users on both sides, the market scale of the platform cannot be expanded, and no new consumers and drug retailers enter the platform to conduct transactions. The development of the platform stagnates and higher profits cannot be obtained, which will eventually lead to the loss of users and profits. Therefore, considering the background of the sustainable development of the pharmaceutical e-commerce platform, this paper does not consider the situation that the platform does not subsidize the users on both sides, but only considers the situation of the platform to choose the subsidy strategy.

In order to simplify the calculation, the rest of this paper meets the inequality, assume:

$$T-V_{C}>0$$

$$R_{C}+\eta_{d}-V_{D}+\eta_{c}>0$$

$$\eta_{c}+P-V_{D}>0\;{4TF-M_{C}M_{D}(R_{C}+\eta }_{d}{-V_{D}+\eta_{c})}^{2}>0$$

Lemma 1 describes the optimal subsidy level of the platform with the goal of maximizing profit under the platform selection subsidy strategy.

### Lemma 1

The optimal level of subsidy for users on both sides of the platform is

$$S_{C}^{*}=\frac{\left[M_{C}M_{D}\left(R_{C}+\eta_{d}-V_{D+}\eta_{c}\right)\left(\eta_{d}-V_{D}+P\right)-2TF\right]\left(T-V_{C}\right)}{4TF-{}_{C}M_{D}{\left(R_{C}+\eta_{d}-V_{D}+\eta_{c}\right)}^{2}}$$ (14)

$$S_{D}^{*}=\frac{FM_{C}(R_{C}+\eta_{d}+\left(T-V_{C}\right)}{4TF-M_{C}M_{D}{\left(R_{C}+\eta_{d}-V_{D}+\eta_{c}\right)}^{2}}$$ (15)

The number of consumers and drug retailers involved in the transaction is:

$$N_{C}^{*}=\frac{2FM_{C}(T-V_{C})}{4TF-M_{C}M_{D}{\left(R_{C}+\eta_{d}-V_{D}+\eta_{c}\right)}^{2}}$$ (16)

$$N_{D}^{*}=\frac{M_{C}M_{D}\left(R_{C}+\eta_{d}-V_{D+}\eta_{c}\right)(T-V_{C})}{4TF-M_{C}M_{D}{\left(R_{C}+\eta_{d}-V_{D}+\eta_{c}\right)}^{2}}$$ (17)

By solving the first derivative, we can get the number of consumers and drug retailers participating in the transaction in equilibrium state. The expressions of *S*_*C*_ and *S*_*D*_ about *N*_*C*_ and *N*_*D*_ can be obtained by combining (12) and (13):

$$S_{C}=\frac{TN_{C}}{M_{C}}-T-\alpha N_{D}+V_{C}$$ (18)

$$S_{D}=\frac{FN_{D}}{M_{D}}-bN_{C}$$ (19)

Substituting Eqs (18) and (19) into the profit function and deriving for *N*_*C*_ and *N*_*D*_ respectively, the following first-order conditions are satisfied:

$$\frac{\partial \pi }{\partial N_{C}}\left(N_{C}^{*},N_{D}^{*}\right)=\frac{\partial \pi }{\partial N_{D}}\left(N_{C}^{*},N_{D}^{*}\right)=0$$ (20)

Then we can get the optimal subsidy level by substituting the number of consumers and drug retailers participating in the transaction into (18), (19). In the two-sided market, only the platform can adopt the subsidy strategy for users on both sides. Therefore, the optimal subsidy level is crucial to adjust the market equilibrium.

Lemma 2 describes the maximum profit of the platform under three subsidy strategies.

### Lemma 2

$$\pi^{*}\left(S_{C}^{*},S_{D}^{*}\right)=\frac{FM_{C}{\left(T-V_{C}\right)}^{2}}{4TF-M_{C}M_{D}{\left(R_{C}+\eta_{d}-V_{D}+\eta_{c}\right)}^{2}}$$ (21)

$$\pi^{*}\left(S_{C}^{*},0\right)=\frac{FM_{C}{\left(T-V_{C}\right)}^{2}+S_{C}N_{C}}{4TF-M_{C}M_{D}{\left(R_{C}+\eta_{d}-V_{b}+\eta_{c}\right)}^{2}}$$ (22)

$$\pi^{*}\left(0,S_{D}^{*}\right)=\frac{FM_{C}{\left(T-V_{C}\right)}^{2}+S_{D}N_{D}}{4TF-M_{C}M_{D}{\left(R_{C}+\eta_{d}-V_{D}+\eta_{c}\right)}^{2}}$$ (23)

The maximum profit of the platform's three subsidy strategies can be obtained by substituting the number of consumers and drug retailers participating in the transaction and the optimal subsidy level in the equilibrium state into the profit function.

According to the above conclusions, it can be concluded that: $S_{C}^{*}N_{C}^{*}>S_{D}^{*}N_{D}^{*}$. Therefore, platform only subsidizes consumers' profits: $\pi^{*}(S_{C}^{*})>$ Platform only subsidizes drug retailers ' profits $\pi^{*}(S_{D}^{*})>$ Platform chooses to subsidize both users' profits $\pi^{*}(S_{C}^{*},S_{D}^{*})$.

In the era of network economy, more and more consumers are shopping online. In such a competitive environment, the number of consumers directly affects the size of the platform's profits. Therefore, the platform adopts a subsidy strategy for consumers, which can not only attract consumers to conduct secondary transactions, but also attract other consumers who have not yet entered the platform, and ultimately achieve the goal of profit maximization.

## Impact of net income and network externality

In Section 4, we compared the size of the profits of three subsidy strategies. More importantly, the equilibrium number of consumers and drug retailers participating in the transaction on the platform directly influences the platform's profits. Nevertheless, there are other factors that affect platform revenue in the real life. One of the most important characteristics of the two-sided market is network externality, which is an important factor affecting retailers' choice of strategies to maximize profits [14]. The more net income consumers get from trading on the platform, the more consumers enter the platform, and the higher the profit of the platform. Therefore, consumer net income is another important factor affecting platform profits [10]. Below we discuss two potential reasons for platform revenue: net income and network externality. When any of these two factors present, the platform benefits will change.

Before we start the discussion, it is important to highlight the value of our previous analysis. First, without the equivalence result established in the basic model, we are unable to understand or even demonstrate the impact of the subsidy strategy chosen by the platform on the platform's own profits. In addition, we may be certain about the impact of various factors on the platform's profit only after the equilibrium profit of platform is determined. The analysis in Section 4 are thus necessary to deliver our messages in this section.

**Proposition 1:** Based on the utility function of consumers, in order to maximize profits, when *M*_*C*_*M*_*D*_(*R*_*C*_+*η*_*d*_−*V*_*D*_+*η*_*c*_*)*(*η*_*c*_+*P*−*V*_*D*_*)*>2*TF*, the platform will choose to subsidize consumers; *R*_*C*_+*η*_*d*_+*V*_*D*_−*η*_*c*_>2*ωP*, the platform may choose to subsidize drug retailers, and subsidies depend on the difference between the platform's drug retailer and the platform.

**Proof:** when *M*_*C*_*M*_*D*_(*R*_*C*_+*η*_*d*_−*V*_*D*_+*η*_*c*_*)*(*η*_*C*_+*P*−*V*_*D*_*)*>2*TF*, $S_{C}^{*}>0$;

When *R*_*C*_+*η*_*d*_+*V*_*D*_−*η*_*c*_>2*ωP*, $S_{D}^{*}>0$; the inequality *R*_*C*_+*η*_*d*_+*V*_*D*_−*η*_*c*_>2*ωP* is rearranged too, in which *ωP*−(1−*ω*)*P* represents the difference between the share of a drug retailer and the platform in each transaction, proposition 1 is proved.

Since the platform will only adopt the subsidy strategy if there is profit, the inequality *α*<*η*_*c*_+*P*−*V*_*D*_ needs to be satisfied when $S_{C}^{*}>0$, where α represents the net income of the consumers participating in each transaction, *η*_*c*_+*P*−*V*_*D*_ represents the total revenue earned by the drug retailer and platform in each transaction. At this point, the net income of consumers is less than the income of drug retailers and platforms, consumers may choose offline channels to buy drugs, the platform if not take appropriate measures, will lose most consumers. In order to ensure that the platform's own profits are not subject to losses, the platform to attract more consumers to enter the platform for trading, choose to subsidize the strategy to attract consumers, increase the tranvolume of platform sactions, and thus improve the platform's own profits.

When the sharing gap is small, the platform can gain more profits directly from drug retailers through the sharing. At the same time, when the drug retailer gives a large proportion of the platform, the revenue of the drug retailer is reduced, and the platform needs to subsidize the drug retailer to avoid a large loss of the drug retailer, thereby promoting the transaction. On the contrary, when the sharing gap is small, the platform cannot get more revenue from the drug retailer through the sharing, and then the platform will give up subsidizing the drug retailer. If drug retailers share a small proportion of the platform, the platform may charge drug retailers transaction fees to cover the cost (i.e. the subsidy level is negative).

**Proposition 2**: When the platform aims at maximizing profits, the platform's subsidy to drug retailers decreases with the increase of drug prices, and the platform's subsidy to consumers increases with the increase of drug prices.

**Proof:** by using the formula (18), (19) to find the first derivative of the retail drug prices can be obtained

$$\frac{\partial S_{C}^{*}}{P}=M_{C}M_{D}\left(R_{C}+\eta_{d}-V_{D}+\eta_{c}\right)$$

$$\frac{\partial S_{C}^{*}}{P}=2\omega FM_{C}\left(V_{C}-T\right)$$

Assumed by the above, *T*−*V*_*C*_>0, *R*_*C*_+*η*_*d*_−*V*_*D*_+*η*_*c*_>0, thus $\frac{\partial S_{C}^{*}}{P}>0,\frac{\partial S_{D}^{*}}{P}<0$, and proposition 2 can be proved.

Specifically, the price of drugs is determined by drug retailers. The high price of drugs in the platform reduces consumers' preference for the platform, and the volume of trading on the platform decreases, resulting in lower profits of the platform. Therefore, the retail price of drugs is equally important for the platform to choose which user to subsidize. When drug retailers have higher prices for drugs, the revenue of drug retailers increases, but the revenue of the platform decreases. At this time, the platform chooses to subsidize consumers or charge corresponding transaction fees to the platform to increase its profits.

**Proposition 3**: When the platform aims at maximizing profits, the number of consumers and drug retailers participating in the transaction increases with the strength of network externalities of users on both sides, and the maximum profit of the platform also varies with the strength of the network.

**Proof:** by using the formula (16), (17), (21) to find the first derivative of the network externality strength parameters can be obtained as follows, that proposition 4 is proved.

$$\frac{\partial N_{C}^{*}}{\partial \eta_{d}}=\frac{\partial N_{C}^{*}}{\partial \eta_{c}}>0$$

$$\frac{\partial N_{D}^{*}}{\partial \eta_{d}}=\frac{\partial N_{D}^{*}}{\partial \eta_{c}}>0$$

$$\frac{\partial \pi^{*}}{\partial \eta_{d}}=\frac{\partial \pi^{*}}{\partial \eta_{c}}>0$$

Through the conclusion, it can be intuitively seen that if the network externality of the consumer is greater, the greater the utility of the new drug retailer entering the platform, the greater the consumer demand, and thus the consumers participating in the transaction. The number will increase, and the more consumers will in turn increase the revenue of drug retailers, and the effectiveness of drug retailers will increase. For the platform, the strength of network externalities increases, the number of consumers and drug retailers involved in the transaction increases, and the volume of platform transactions increases, so the platform can obtain more revenue. At this time, the platform to adopt a subsidy strategy, will attract more users on both sides to join the platform, although more on both sides of the user into the platform means that the platform will pay more costs, but the platform can get more revenue from the larger market size and effective subsidy strategy.

**Proposition 4:** When the strength of network externality brought by drug retailers to consumers is greater than that brought by drug retailers to consumers, that is, when *η*_*d*_>*η*_*c*_, consumer's net income *R*_*C*_+*η*_*d*_−*P* increases, the platform chooses to subsidize drug retailers; on the contrary, when *η*_*d*_<*η*_*c*_, total revenue *η*_*c*_*+P*−*V*_*D*_ of the platform and drug retailers increased, the platform chooses to subsidize consumers.

Proposition 4 shows that when the strength of network externalities brought by drug retailers to consumers is greater than that brought by drug retailers to consumers, the platform can attract more consumers to participate in the transaction through subsidizing drug retailers, thus expanding the scale of the platform market. When consumers bring drug retailers network externality strength is greater than the drug retailers are bringing consumers the strength of network externality, at this point $S_{D}^{*}<0$, meaning that if platform continue to subsidise drug retailers will cause lower profits, so the platform that subsidize consumers to attract more consumers into the platform to participate in the transaction, will get a higher income.

Based on the above assumptions, the first derivative of the variable parameters is obtained for each optimal solution, and the relationship between the variables can be obtained as shown in the Table 4.

**Table 4:** Monotonicity between optimal solution and variable parameters.

Variable Optimal solution	T	F	M_C_	M_C_	P
**N**_C_*	↑	↑	↓	---	---
**N**_D_*	↑	↑	↓	↑	---
**S**_C_*	↓	↓	↑	?	↑
**S**_D_*	↑	↑	?	↓	↓
π*	↑	↑	↑	↑	---

(↑Monotonically increasing; ↓ Monotonically decreasing;—Unrelated; ? Undetermined; * Optimal solution)

As can be seen from the table, when the drug sales price increases, the platform tends to increase subsidies to consumers, reduce subsidies to drug retailers, and even charge drug retailers a certain transaction fee. At this point, drug retailers have obtained higher returns by the sale of high-priced drugs through the platform. The platform should choose to increase subsidies to consumers in order to increase the utility of consumers, so as to avoid the decline in the number of consumers caused by high prices, and the profits that the platform makes through subsidies. The profits lost by the platform through subsidies can be compensated by charging transaction fees to drug retailers.

As consumers and drug retailers gain more utility from platform transactions, the platform can make more profits due to the network externalities of the two-sided market, which will attract more and more users from both sides to join the platform for trading.

Either way, when the number of potential users increase, the platform will reduce the level of subsidy to the other users, because the larger the number of users on one side, the greater the number of users joining the platform and the greater profits for users on the other side, the platform can benefit directly from users who make more profits, there is no need to subsidize it to maximize profits.

## Conclusions

From real life, we can see that in the early stage of platform development, platform enterprises in the two-sided market generally adopt the subsidy strategy of "burning money", subsidize users on one side of the platform to make them join the platform to trade, through the participation of subsidized users, improve the interests of the other side users and the development of the platform. As an entry point, this paper based on the pharmaceutical e-commerce platform generally adopted "full preferential reduction" and " discount promotion " and so on the objective phenomenon of strategy, build the consideration on both sides of the platform of user utility and subsidy level for the two-sided dynamic game model, aims to research platform for users on both sides of the optimal level of subsidies and platform to obtain the maximum profit.

In this study, we present a game-theoretic study featuring network externality to investigate three subsidy strategies in platform operation.

Some distinguished contributions of this study are as follows:

1) The platform chooses to subsidize consumers in order to maximize the profits of the platform, only if the net income from consumer participation in each transaction is lower than the total gains made by drug retailers and platforms in each transaction.

2) When drug retailers give the platform a larger share, the platform needs to subsidize drug retailers to avoid a massive loss of drug retailers, which in turn facilitates trading. On the contrary, when the gap is smaller, the platform cannot get more revenue by dividing it from the drug retailer side, and the platform will abandon subsidies to drug retailers.

3) If drug retailers bring less network externalities to consumers, or when consumers bring greater network externalities to drug retailers, the platform tends to choose to subsidize consumers, and if consumers bring less network externalities to drug retailers, or when drug retailers bring greater network externalities to consumers, the platform tends to choose to subsidize drug retailers.

4) When the platform aims to maximize profits, the number of consumers involved in the transaction and the number of drug retailers involved in the transaction increase with the increase of the network externalities of the users on both sides; the maximum profit of the platform increases with the increase of the network externality intensity.

5) When the price of drug sales increases, the platform tends to increase subsidies to consumers, reduce subsidies to drug retailers, and even charge drug retailers a certain amount of transaction fees.

This study is constrained to the subsidy strategy of monopoly platform, but in the actual market competition, there are many medical e-commerce platforms using subsidy strategy for competitive games and users can choose to operate on multiple platforms. Future work will focus on multi-platform competition, user multilateral attributes and other research issues.

## References

[1] Armstrong M. Competition in two-sided markets. The RAND Journal of Economics.2006; 37(3):668–691.

[2] Armstrong M, Wright J. Two-sided markets, competitive bottlenecks and exclusive contracts. Economic Theory. 2007; 2:358–380.

[3] Bilotkach V, Rupp N. G. Buyer subsidies in two-sided markets: Evidence from online travel agents. The Economics of International Airline Transport. 2012; 4:339–374.

[4] Borcka R, Wrede M. Subsidies for intracity and intercity commuting. Journal of Urban Economics. 2009 7; 66(1): 25–32.

[5] Caillaud B, Jullien B. Chicken & egg: Competition among intermediation service providers. The RAND Journal of Economics. 2003 2; 34(2):309–328.

[6] Chao Y, Derdenger T. Mixed bundling in two-sided markets in the presence of Installed base effects. Management Science. 2013 8; 59(8):1904–1926.

[7] Chen Z, Li J, Liu Z, Zheng Z. Incentive contracts for capacity restoration under risk of supply disruption. IEEE Transactions on Engineering Management. 2018 7:1–17.

[8] CMH. Pharmaceutical retail terminal market scale grows steadily [EB/OL].2017; 12, 18. Retrieved from: http://www.so.com/link?m=aVomr%2BJQVYq2UDVWcZQ4VWt4Z%2F5AL1AOgT853LCpXxoEJRHFV9SmPhjdLyyi0GKHFOA9e7nANHrap4VwvmCOSfvHuR9GcgyxTom1Po6CDM5u6TgTGz8MH%2BLviorS%2BScA%2FDeVCdhB8DQhgdV9F4VdVYwECpC

[9] Dou G, Dou G, Xu X. One-side value-added service investment and pricing strategies for a two-sided platform. International Journal of Production Research. 2016 2; 54(13):1–14.

[10] Emmanuel F, Hagiu A. Strategic interactions in two-sided market oligopolies. Ssrn Electronic Journal. 2008 7:8–11.

[11] Fan R, Dong L. The dynamic analysis and simulation of government subsidy strategies in low-carbon diffusion considering the behavior of heterogeneous agents. Energy Policy. 2018 6; 117:252–262.

[12] Hagiu A. Pricing and commitment by two-sided plat-forms. The Rand Journal o fEconomics. 2006 9; 37:720–737.

[13] Hagiu A, Halaburda H. Information and two-sided platform profits. International Journal of Industrial Organization. 2014; 34:25–35.

[14] Howell B. Unveiling‘invisible hands’: Competition in two-sided health care markets. Social Science Electronic Publishing 2006: 35–35.

[15] Jin C, Wang X, Junmei L. Analysis on the optimal subsidy strategy of government in green supply chain. Advanced Materials Research. 2011 4; 224: 147–151.

[16] Kelly B. D. The pass-through of subsidies to price. Journal of World Trade. 2014 4; 48(2): 295–295.

[17] Kim S. How can we make a socially optimal large-scale media platform? Analysis of a monopolistic Internet media platform using two-sided market theory. Telecommunications Policy. 2016 9; 40(9): 899–918.

[18] Lin M, Li S, Whinston A. B. Innovation and price competition in a two-sided market. Journal of Management Information Systems. 2011 10; 28(2): 171–202.

[19] Lin M, Wu R, Zhou W. Endogenous network effects, platform pricing and market liquidity. Social Science Electronic Publishing. 2014; 18: 424.

[20] Ma J, Sui X, Li L. Measurement on the Complexity Entropy of dynamic game models for Innovative enterprises under Two kinds of government subsidies. Entropy. 2016 11; 18(12): 424.

[21] Muzellec L, Ronteau S, Lambkin M. Two-sided internet platforms: A business model lifecycle perspective. Industrial Marketing Management. 2015 2; 45:139–150.

[22] Richards T. J., & Hamilton S. F. Food waste in the sharing economy. Food Policy 2018 2, 75: 109–123.

[23] Rochet J. C, Tirole J. Two-sided markets: An overview. Toulouse. 2004; 51(11):233–260.

[24] Rochet J. C, Tirole J. Two-sided markets: A progress report. The RAND Journal of Economics.2006 9; 37(3): 645–667.

[25] Shih M, Wu T, Wei H. Unlicensed LTE pricing for tiered content delivery and heterogeneous user access. IEEE Transactions on Mobile Computing 2018 4; 99, 1.

[26] Wang Y, Tang J, Jin Q, Ma J. On studying business models in mobile social networks based on two-sided market. The Journal of Supercomputing. 2014 12; 70(3):1297–1317.

[27] Wang P, Zhang R, Tang B, Yuan M. Pricing model and influential factors analysis of drug trade service platform. LISS 2013 Dec: Proceedings of 3rd International Conference on Logistics, Informatics and Service Science: 221–226.

[28] Wei M. M, Yao T, Jiang B, Young S. T. Profit seeking vs. survival seeking: An analytical study of supplier's behavior and buyer's subsidy strategy. Production and Operations Management. 2013 3; 22(2): 269–282.

[29] Zhang N. Tapio Levä, Heikki hämmäinen. Value networks and two-sided markets of internet content delivery. Telecommunications Policy. 2014 5; 38(5/6): 460–472.

[30] Zhang Q. Research on Medicine E-business Platform Model Based on Value-chain and Value-net Theory. International Conference on Wireless Communications, Networking and Mobile Computing 2007 Sep.

